# Evaluation of the Intel Xeon Phi 7120 and NVIDIA K80 as accelerators for two-dimensional panel codes

**DOI:** 10.1371/journal.pone.0178156

**Published:** 2017-06-05

**Authors:** Lukas Einkemmer

**Affiliations:** Department of Mathematics, University of Innsbruck, Innsbruck, Austria; Technische Universitat Darmstadt, GERMANY

## Abstract

To optimize the geometry of airfoils for a specific application is an important engineering problem. In this context genetic algorithms have enjoyed some success as they are able to explore the search space without getting stuck in local optima. However, these algorithms require the computation of aerodynamic properties for a significant number of airfoil geometries. Consequently, for low-speed aerodynamics, panel methods are most often used as the inner solver. In this paper we evaluate the performance of such an optimization algorithm on modern accelerators (more specifically, the Intel Xeon Phi 7120 and the NVIDIA K80). For that purpose, we have implemented an optimized version of the algorithm on the CPU and Xeon Phi (based on OpenMP, vectorization, and the Intel MKL library) and on the GPU (based on CUDA and the MAGMA library). We present timing results for all codes and discuss the similarities and differences between the three implementations. Overall, we observe a speedup of approximately 2.5 for adding an Intel Xeon Phi 7120 to a dual socket workstation and a speedup between 3.4 and 3.8 for adding a NVIDIA K80 to a dual socket workstation.

## 1 Introduction

Numerical simulations are routinely used in applications to predict the properties of fluid flow over a solid geometry. Such applications range from the design and analysis of aircrafts to constructing more efficient wind turbines. In this context, a large number of different models and numerical methods have been developed to efficiently compute aerodynamic quantities such as lift and drag. It is generally believed that the compressible Navier—Stokes system is able to represent the physics that is encountered in such systems faithfully. However, even for moderate Reynolds numbers, turbulent motion is only dissipated at very small spatial scales. This forces an extremely fine space discretization and renders the numerical solution of the time dependent Navier—Stokes system intractable in all but a very selective class of applications (this approach is usually referred to as DNS or direct numerical simulation). Consequently a hierarchy of reduced models has been developed that are computationally more efficient. Even though methods such as RANS (Reynolds-averaged Navier—Stokes) and LES (Large eddy simulations) are routinely employed to perform aerodynamics simulations, these simulations can still require days or even weeks to complete.

In this work the goal is to develop a computer program that is able find an ideal airfoil geometry given a target function (for example, this target could be to maximize the lift-to-drag ratio). This is a nonlinear optimization problem as the geometry is the parameter under consideration. In addition, the large number of maxima found in these problems renders traditional optimization algorithms ineffective. In recent years, genetic algorithms have enjoyed some success (see, for example, [1–3]). However, their application yields a new computational challenge as they require the computation of thousands or even hundred thousands of different airfoil configuration. Consequently, even RANS or LES methods are computationally prohibitive as the inner solver in such an optimization algorithm.

In this paper we will restrict our attention to low-speed aerodynamics. That is, we assume that the flow under consideration is slow compared to the speed of sound. These conditions are present in a wide range of applications (for example, unmanned aerial vehicles and wind turbines). Since the flow is slow compared to the speed of sound it is justified to neglect compressible effects. In addition, we make the assumption that the flow is irrotational. In this case the Navier—Stokes equations reduce to Laplace’s equation. One should note that a direct solution of Laplace’s equation would result in a body with zero lift. However, by imposing an additional constraint, the so-called Kutta condition, this simple model yields very accurate results in its regime of validity (even for lifting bodies such as airfoils, rotor blades, or fins). In addition, many phenomenological corrections have been developed that are able to extend the range of validity of this simplified model considerably.

In principle, any numerical method can be used to solve Laplace’s equation together with the Kutta condition. However, since we are usually interested in the fluid flow outside of a solid body, so-called panel methods (or boundary element methods) have become the standard approach. The advantage of such a method is that only the boundary has to be discretized. This implies that for a two-dimensional flow only a linear system in a single dimension has to be solved (although the corresponding matrix is no longer sparse). In addition, no error is made by introducing an artificial boundary faraway from the dynamics of interest. On modern computers a good implementation is able to compute, for example, the flow over an airfoil in less than a few tens of milliseconds (although this has not always been true in the past). Especially in the early days of computational fluid dynamics, performing such simulations was the only way to obtain results in a reasonable time. As a consequence, sophisticated software packages (such as Xfoil [4]) have been developed that are still used in current aerodynamics research (see, for example, [2, 5–7]).

The main advantage of panel methods is that they are computationally cheap and that fact makes them ideally suited as the inner solver in an optimization algorithm. In addition, they are able to faithfully reproduce the relevant aerodynamic quantities for low-speed aerodynamics [8].

The described optimization problem lends itself well to parallelization. As such it can potentially profit significantly from accelerators such as graphic processing units (GPUs) or the Intel Xeon Phi. In fact, some papers have been published that implement panel methods on GPUs (see, for example, the work conducted in [9–12]). However, most of the literature focuses on the three dimensional case. where the linear solve dominates the performance of the algorithm. As we will see in section 3 this is not true for the two-dimensional problem. In addition, speedups between one and two orders of magnitude are routinely reported [9, 10, 12, 13]. However, since the hardware characteristics of the central processing unit (CPU) and the graphic processing unit (GPU) do not admit such a large difference in performance, it has to be concluded that the performance on the GPU has been compared to a CPU implementation that is not very well optimized. In this context it should be noted that CPU based system now include tens of cores and thus parallelization (and vectorization) is vital in order to obtain optimal performance on those systems as well.

The purpose of the present work is therefore to parallelize the optimization problem described above (of which the panel method is the computationally most demanding part) on both traditional CPU based systems as well as on the GPU and to compare their performance. In addition, we consider a parallel implementation on the Intel Xeon Phi. The Xeon Phi is an accelerator (which is added as an expansion card similar to a GPU) based on the x86 architecture. As such this platform promises to accelerate the computation while still enabling the use of the same development tools (and ideally the same code) as on the CPU. For example, to parallelize code for the Xeon Phi OpenMP is usually employed. We compare the performance of the Xeon Phi to the implementation on the CPU and the GPU. Furthermore, we will consider the parallelization to multiple GPUs which poses additional challenges.

The numerical algorithm used in this paper is described in more detail in section 2. In section 3 we then discuss the performance characteristics of the algorithm, the hardware used, and the general idea of the implementation. The timing results and details of the specific implementation under consideration are then presented in sections 4 (single GPU), 5 (Xeon Phi), and 6 (two GPU setup). Finally, we conclude in section 7.

## 2 Numerical algorithm

Panel methods are a type of boundary element methods. In order to remedy the deficiency of Laplace’s equation to describe the airflow over lifting bodies, they are supplemented by the empirically derived Kutta condition. This model, in many instances, gives a good description of lifting flow over solid bodies for low speed aerodynamics [8]. In the following, we will limit ourselves to two-dimensional flows over wing cross sections (so-called airfoils).

**Fig 1:**
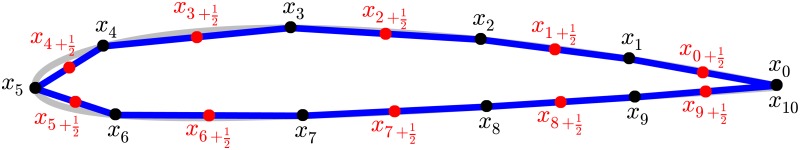
The discretized geometry of the NACA 2412 airfoil is shown (for the purpose of illustration a very coarse discretization with *n* = 10 is employed). The control points are shown in red and the exact geometry is outlined in gray.

The geometry of the problem is given by a sequence of points $x_{0},x_{1},\ldots ,x_{n}\in R^{2}$ that represent the discretization of an airfoil ∂Ω. We assume that ***x***_0_ is located at the trailing edge and that ***x***_*n*_ = ***x***_0_ holds true. This setup is illustrated in Fig 1.

The goal of the numerical method is to compute an approximation to the solution of Laplace’s equation in $R^{2}\setminus \Omega$. This solution, henceforth denoted by *φ*, physically represents a stream function and encodes all properties of a two-dimensional incompressible flow. For example, the velocity of the flow can be computed by *v*_1_ = ∂_*y*_*φ* and *v*_2_ = −∂_*x*_*φ*, where *v*_1_ is the velocity in the *x*-direction and *v*_2_ is the velocity in the *y*-direction. Consequently the velocity vector ***v*** is expressed as ***v*** = (*v*_1_, *v*_2_)^T^.

Panel methods represent the solution as a superposition of translations of the fundamental solution (which by itself is a solution of Laplace’s equation everywhere except at zero)

$$\begin{array}{c} \phi \left(x\right)=-\frac{1}{2\pi }\log\left|x\right|. \end{array}$$

and the global flow that is imposed far away from the airfoil. Thus, the solution *φ*(***x***) will be written as

$$\begin{array}{c} \varphi \left(x\right)=\int_{\partial \Omega }\gamma \left(s\right)\phi \left(x-s\right)\;ds+\phi_{v}\left(x\right), \end{array}$$

where *γ*(***s***) is the coefficient in the superposition. The stream function of the global flow with velocity ***v*** = (*v*_1_, *v*_2_)^T^ is given by

$$\begin{array}{c} \phi_{v}\left(x\right)=v_{1}y-v_{2}x=v_{\infty }y\cos\alpha -v_{\infty }x\sin\alpha , \end{array}$$

where *v*_∞_ = |***v***| is the speed of the global flow and the parameter *α* is called the angle of attack (note that ***v*** = *v*_∞_(cos*α*, sin*α*)^T^). Laplace’s equation is subject to the boundary condition

$$\begin{array}{c} {\varphi |}_{\partial \Omega }=C, \end{array}$$

which enforces that no fluid can move perpendicular to the wall. Note that value of *C* will be determined as part of the numerical solution. We discretize this ansatz by assuming that the vortex strength *γ*(***s***) is constant on each panel. For a panel from ***x***_*i*_ to ***x***_*i*+1_ with vortex strength *γ*_*i*_ this yields

$$\begin{array}{cc} F_{i}\left(x\right) & =\int_{x_{i}}^{x_{i+1}}\gamma_{i}\phi \left(x-s\right)\;ds\\ & =\frac{\gamma_{i}}{2\pi }\frac{1}{|h_{i}|}\left[\frac{1}{2}\left\langle x-x_{i},h_{i}\right\rangle \log{\left|x-x_{i}\right|}^{2}\right.\\ & \;-\frac{1}{2}\left\langle x-x_{i+1},h_{i}\right\rangle \log{\left|x-x_{i+1}\right|}^{2}\\ & \;-I\text{arctan2}(I,\left\langle x-x_{i},h_{i}\right\rangle )\\ & \;\left.+I\text{arctan2}\left(I,\left\langle x-x_{i+1},h_{i}\right\rangle \right)-{\left|h_{i}\right|}^{2}\right] \end{array}$$

where $I=\langle h_{i}^{⊥},x-x_{i}\rangle$, ***h***_*i*_ = ***x***_*i*+1_ − ***x***_*i*_, and $h_{i}^{⊥}$ is the outward pointing vector that is orthogonal to ***h***_*i*_ and satisfies $|h_{i}^{⊥}\left|=\right|h_{i}|$. We have used 〈⋅, ⋅〉 to denote the dot product. The boundary condition is enforced at the control points (i.e., at ***x***_*i*+1/2_ = (***x***_*i*+1_ + ***x***_*i*_)/2). This yields an underdetermined system of linear equations

$$\begin{array}{c} -\sum_{i=0}^{n-1}F_{i}\left(x_{j+1/2}\right)+C=\sum_{i=0}^{n-1}A_{ji}\gamma_{i}+C=\phi_{v}\left(x_{j+1/2}\right) \end{array}$$

which we supplement by the Kutta condition

$$\begin{array}{c} \gamma_{0}=-\gamma_{n-1}. \end{array}$$

In stating the Kutta condition we have assumed that the variables are ordered such that the trailing edge is located at ***x***_0_ = ***x***_*n*_. This, in total, gives us *n* equations for the *n* unknowns *γ*_0_, …, *γ*_*n*−2_ and *C*.

While the present numerical scheme yields good predictions for the lift coefficient, it gives completely wrong results for the drag coefficient. This is to be expected as drag is a viscous effect. However, a range of phenomenological corrections has been developed that, for attached flows, are able to predict the drag coefficient based on the inviscid solution. In our code we have implemented Thwaites’ method (see, for example, [14, 15]) in order to perform a viscosity correction.

**Fig 2:**
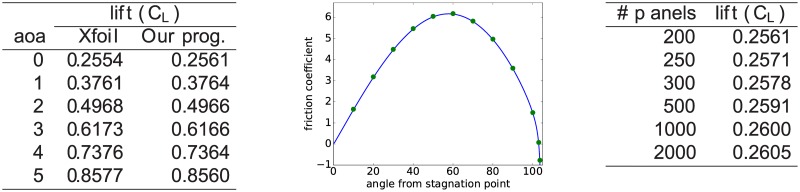
On the left the lift of an NACA 2412 airfoil as predicted by our program is compared to Xfoil. In the middle the drag for a circular cylinder as predicted by our program (blue line) is compared to the analytic solution of Thwaites’ method (the green points correspond to some values of the analytic solution which, for example, have been tabulated in [16]). On the right the dependence of the predicted lift coefficient on the number of panels used is investigated.

To validate the implementation we have compared the results for the lift obtained by our program to Xfoil. As can be seen from Fig 2 there is excellent agreement (the difference between the two programs is well below 1%). Unfortunately, such a comparison is not possible for the drag as the models used for viscosity correction are different in the two programs. However, for Thwaites’ method an analytic solution can be obtained for the drag over a circular cylinder. The comparison of our program with this analytic solution is shown in Fig 2. We once again observe excellent agreement. Finally, we have increased the number of panels used to discretize the airfoil. We find that it is generally sufficient to use 200 to 300 panels in order to obtain an error on the order of 1%. This is certainly sufficient as neither the accuracy of the model used nor practical considerations would justify using more precision.

As has been outlined in the introduction, traditional optimization algorithms often get stuck in local minima and are thus unsuitable for the problem of interest here. Consequently the performance of a number of global search algorithms has been investigated. This includes genetic algorithms, simulated annealing, CRSA (controlled random search algorithms), etc. Among these methods genetic algorithms have been recognized as one of the best performing options (see, for example, [17]) and have been extensively employed in a variety of applications [1–3, 7]. Therefore, we employ a genetic algorithms to perform the optimization. The first step is to choose a parametrization of the geometry. In the language of genetic optimization this is called the representation of the genome. In our implementation we describe the geometry by a B-spline curve. The location of the B-spline knot points (ordered from the trailing edge on the upper part of the airfoil to the trailing edge on the lower airfoil) form the representation of the genome used in the implementation. The genetic algorithm then proceeds as follows

1. Initialize a population of airfoil geometries (individuals) at random. That is, initialize each individual by choosing the B-spline knots at random (within reasonable bounds).
2. Evaluate the target (fitness) function for each individual using the panel method described above.
3. Select promising individuals from the population (i.e. individuals with a high fitness value).
4. Combine pairs of promising individuals (parents) in order to generate individuals for the next generation (children).
5. Perform, with a certain probability, a random mutation of a given individual.
6. Go to 2.

The purpose of the selection step is to favor the propagation of fitter individuals. The rational behind this bias is that the combination of features from two good individuals might result in an individual with even better fitness. In our implementation we employ tournament selection. That is, we choose *k* individuals from the population at random. The best individual (the individual with the highest fitness) within that group is then selected with probability *p*. The second best individual with probability *p*(1 − *p*), and so on. Two individuals, selected in the manner described, are then combined into two children by a crossover operation. The crossover is performed by choosing (at random) a position in the genome (the list of B-spline coefficients) and all coefficients prior to that point are taken from the first parent while all coefficients starting at that point are taken from the second parent (this is usually referred to as one-point crossover). By reversing the order of the two parents, we obtain a second child. This procedure is repeated until the new generation has the desired number of individuals. The final step in the algorithm is then to perform so-called mutations. That is, for each individual there is a certain probability that we perturb one of its B-spline coefficients. Mutation is crucial in order to prevent the premature convergence of the algorithm. If the probability of mutation is too low, the algorithm can easily get stuck in a local maximum (which we strive to avoid). For more details on genetic algorithms we refer the reader to [18].

**Fig 3:**
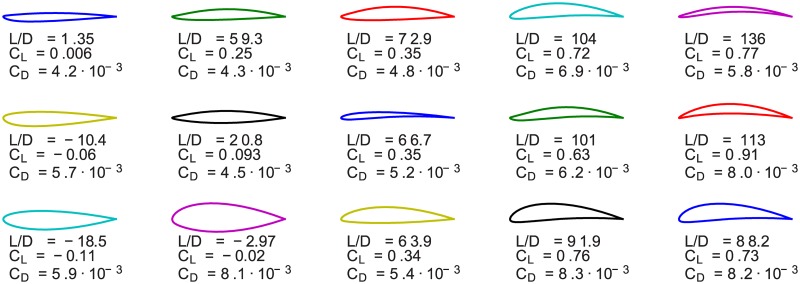
Three airfoils for generation 1, 2, 3, 6, and 7 of the genetic optimization algorithm are shown. The algorithm proceeds from the left to the right and each column represents a distinct generation. We show the best classes of airfoils (according to the lift-to-drag ratio) for a specific generation. The population size is equal to 1000.

**Fig 4:**
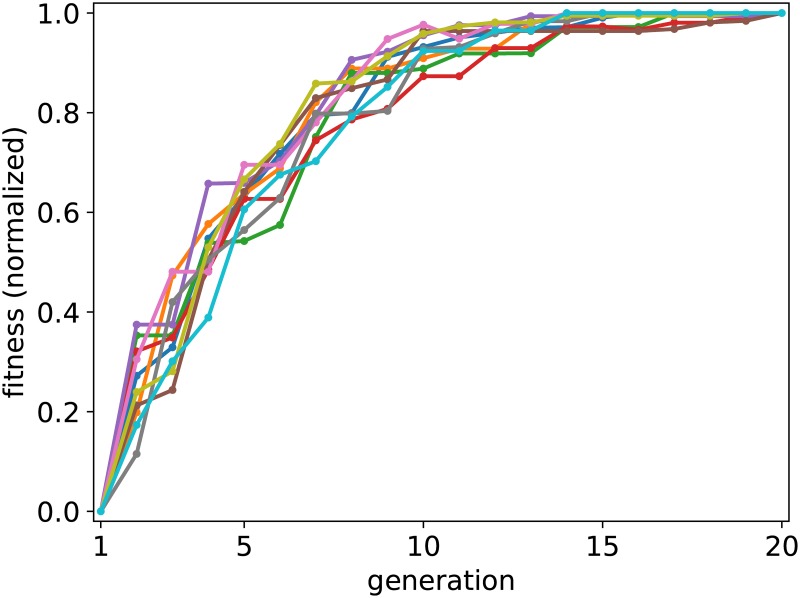
The fitness of the population (normalized to the overall best individual) as a function of the generation in the genetic algorithm is shown for ten different (random) initial configurations.

In Fig 3 the evolution of the optimization algorithm is shown. In this simulation the fitness function is proportional to the lift-to-drag ratio at zero angle of attack. The lift and drag coefficients stated in the figure are computed using Xfoil (as opposed to using the output of our simulation). This is done in order to validate that our code performs as expected. In addition, we have investigated the convergence of the genetic algorithm as a function of the number of generations computed. The result is shown in Fig 4.

## 3 Computational considerations

The numerical implementation of the above algorithm requires two parts of significant computational effort. First, the system of linear equations has to be assembled which requires $O(n^{2})$ operations but involves the (expensive) evaluation of two logarithms and two arctan2 functions for each panel. Second, the solution of the linear system of equations is usually done by an LU decomposition and thus involves $\frac{2}{3}n^{3}$ operations. In practice *n* is often between 100 and 300. In this regime both parts of the algorithm require substantial computational effort.

In the following we will consider the CPU, Xeon Phi, and GPU configuration listed in Table 1. These will be used for all the numerical simulations and all the performance measurements conducted in this paper. The corresponding (peak) performance characteristics with respect to single and double precision arithmetics and the theoretical attainable memory bandwidth are listed in Table 1. All of these components are part of a single dual socket workstation.

**Table 1:** Hardware characteristics of the dual socket workstation used in the numerical simulations. Peak arithmetic performance for single and double precision and the theoretically attainable memory bandwidth are listed.

	TFlops/s		
	double	single	GB/s
1x E5-2630 v3	0.3	0.6	59
2x E5-2630 v3	0.6	1.2	59
1x Xeon Phi 7120	1.2	2.4	352
0.5x K80	1.5	4.4	240
1x K80	2.9	8.7	480

Some fairly representative single and double precision timing results are collected in Table 2. These results point the clear picture that on the CPU assembling the matrix is between 2.5 and 3.5 times more expensive compared to solving the resulting linear systems. Thus, on the CPU the assembly actually dictates the performance of the algorithm to a large extend. This situation is reversed for both the Xeon Phi 7120 and the K80 GPU. For the Xeon Phi 7120 the assembly step is by approximately a factor of two faster compared to the two CPUs. Since assembly is an extremely compute bound problem and giving the similarities of the two architectures, this gain is expected based on the factor of two difference in the theoretical arithmetic performance. On the other hand, one half (note that the NVIDIA K80 is a single expansion card that includes two identical GPUs each with its own separate memory) of the K80 outperforms the same two CPUs by a factor of approximately 5 and the Xeon Phi 7120 by approximately a factor of 3. Note that the GPU architecture includes a number of so-called multi-function units (MUFU) per streaming mutiprocessor. These are used to accelerate the computation of certain transcendental functions. Let us emphasize that double precision support of the multi-function units is limited. However, double precision support for the reciprocal (which is used in the assembly code generated for both the log and the atan2 function) is available.

**Table 2:** Time in seconds that is required to perform the assembly and linear solver step in our panel code. In the simulation 4000 candidate solutions (airfoil geometries) are optimized using a genetic algorithm with 10 generations. Each geometry is discretized using 200 points. For the linear solve we use the Intel MKL 2015 library (on the CPU and Intel Xeon Phi 7120) and the MAGMA 1.7.0 linear algebra library on the NVIDIA K80. All measured times are in units of seconds.

**single precision**			
	Assembly	Solve	Total
E5-2630 v3	4.93	1.68	6.61
2x E5-2630 v3	2.70	1.00	3.70
Phi 7120	1.35	3.60	4.95
0.5x K80	0.46	3.70	4.16
**double precision**			
	Assembly	Solve	Total
E5-2630 v3	9.26	2.80	12.05
2x E5-2630 v3	5.11	1.91	7.01
Phi 7120	2.69	4.72	7.41
0.5x K80	0.79	4.42	5.21

The performance of the linear solver is relatively poor on both the Xeon Phi 7120 as well as on the NVIDIA K80. Note that in our application we are not interested in solving large linear systems (for which both of these libraries provide excellent performance) but in solving a large number of relatively small linear systems. In this situation the linear solve is not necessarily compute bound (this is particularly true on architectures with a high flop/byte ratio). In addition, the irregular memory access patterns encountered in this algorithm also favor systems with more elaborate caches. Let us note that it might be possible to improve the performance of the linear solve on the Xeon Phi. In fact, some research has already been conducted in this direction (see, for example, [19, 20]). The same is presumably true for the GPU.

It thus seems that neither the CPU nor accelerators are ideally suited for the problem under consideration. However, since the accelerators are very efficient in the assembly step and the CPUs are very efficient in the linear solve step, the hope is that a hybrid algorithm that uses both platforms can succeed in obtaining a significant speedup compared to a CPU only implementation. The difficulty in this approach is that a large amount of data has to be transferred over the (relatively) slow PCIe bus. In the problem under consideration this means that all the assembled matrices have to be transferred from the accelerator to the CPU. Clearly, if such a scheme is to be successful some strategy has to be employed to mitigate this communication overhead. To present an efficient implementation and the corresponding benchmark results for both the Intel Xeon Phi 7120 and for the NVIDIA K80 is the purpose of the remainder of this paper.

To conclude this section let us mention the development tools used on the respective platforms. On the CPU and the Xeon Phi we employ the Intel C++ compiler and, in order to perform the parallelization, OpenMP. To solve the linear system on the CPU the Intel MKL library is used (which provides highly optimized LAPACK routines). For the GPU implementation we employ the CUDA framework and, for the multiple GPU implementation, the MAGMA linear algebra library.

## 4 GPU implementation

**Fig 5:**
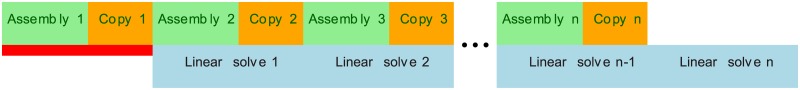
This figure shows a communication hiding pattern that interleaves the assembly (green; on the GPU) and copy (orange; data transfer from the GPU to the CPU) with the linear solve (blue; on the CPU). The red areas constitute the remaining overhead that decreases as we divide our problem into more and more slices.

In this section we will consider an implementation where the assembly of the matrix is conducted exclusively on the GPU and the linear solves are performed exclusively on the CPU. This requires the transfer of a large number of matrices in each step from the GPU to the CPU. Timing results indicate that the run time of the assembly step (on the GPU) together with the required transfer of data (from the CPU to the GPU) is comparable or smaller than the time it takes to perform the linear solve (on the CPU). Thus, to hide the communication overhead, we interleave the assembly and transfer operation with the linear solves on the CPU. This is possible since, in principle, the assembly step can be computed independently for each individual in the population. There is, however, a computational advantage in aggregating multiple such operations together in a single slice. Therefore, we divide the population into (usually between 5 and 20) subpopulation. Each of these slices of the population is then assembled and send to the CPU. While the CPU is conducting the linear solve another slice is assembled on the GPU. This approach is illustrated in Fig 5. In the implementation CUDA streams are used to asynchronously compute on the GPU as well as to asynchronously transfer data from the GPU to the CPU. It is also possible to interleave the assembly and copy operations. However, for the GPU we found that this does not result in an increase in performance. Thus, for the remainder of this section we will restrict ourselves to the two-way interleave scheme illustrated in Fig 5.

Note that the overhead of this approach decreases as we increase the number of slices our problem is partitioned into. However, since the individual problems become smaller and smaller, overhead inherent in the different parts of the algorithm becomes more pronounced. Therefore, a compromise has to be made. In general, between 10 and 20 slices seems to yield near optimal performance in most circumstances.

The timing results are given in Table 3. We observe a speedup of 3 (single precision) and 2.9 (double precision) for adding a single K80 to the dual socket workstation. Although even a naive implementation (i.e., doing the assembly, the data transfer, and the linear solve in sequential order) results in some speedup, the communication hiding scheme employed contributes significantly to the performance of the implementation. In the case of a single socket workstation the observed speedup is approximately 3.6 (single precision) and 4.0 (double precision).

**Table 3:** Timing results for the hybrid algorithm (one-half NVIDIA K80+CPU) illustrated in Fig 5. The wall time (W), the time required to assemble the system (A), the time required for the linear solves (L) and the overhead due to offloading to the GPU (O) are shown. Note that for the GPU implementation the time required by the linear solve (which is done on the CPU) always dominates the total runtime. Thus, we have W = L + O. The number of slices that yield the optimal run time are shown in bold. All measured times are in units of seconds. In addition, the standard deviation determined from 20 repetitions of the simulation is shown next to the wall time.

**single precision**						
Hardware	slices	W	L	O	A	speedup
CPU	–	6.61±0.01	1.68	–	4.93	–
GPU+CPU	1	2.68±0.05	1.66	1.02	0.46	2.46
	5	1.95±0.02	1.71	0.24	0.46	3.39
	10	1.90±0.02	1.75	0.15	0.46	3.48
	15	1.86±0.02	1.75	0.12	0.47	3.55
	**20**	**1.84±0.02**	**1.73**	**0.10**	**0.47**	**3.60**
2xCPU	–	3.70±0.03	1.00	–	2.70	–
GPU+2xCPU	1	2.06±0.03	1.03	1.03	0.46	1.80
	5	1.32±0.02	1.08	0.24	0.46	2.81
	10	1.24±0.03	1.10	0.15	0.46	2.97
	15	1.24±0.02	1.12	0.12	0.46	2.99
	**20**	**1.22±0.02**	**1.12**	**0.10**	**0.47**	**3.03**
**double precision**						
Hardware	slices	W	L	O	A	speedup
CPU	–	12.05±0.01	2.80	–	9.26	–
GPU+CPU	1	4.80±0.04	2.90	1.90	0.77	2.51
	5	3.39±0.06	2.93	0.47	0.77	3.55
	10	3.09±0.07	2.82	0.27	0.77	3.89
	**15**	**2.99±0.09**	**2.77**	**0.22**	**0.78**	**4.03**
	20	3.08±0.07	2.88	0.20	0.78	3.91
2xCPU	–	7.01±0.01	1.91	–	5.11	–
GPU+2xCPU	1	3.93±0.03	2.02	1.91	0.77	1.79
	5	2.59±0.03	2.12	0.47	0.77	2.70
	10	2.45±0.02	2.15	0.29	0.78	2.86
	**15**	**2.44±0.02**	**2.20**	**0.24**	**0.78**	**2.88**
	20	2.44±0.03	2.23	0.21	0.78	2.87

The overhead in this implementation can be partitioned into two parts:

- As we partition our problem into more and more slices the performance of the linear solver on the CPU decreases. This is a consequence of the overhead required for the asynchronous data transfer to the GPU as well as the overhead that is incurred in decreasing the batch size for the linear solver. In the numerical simulations conducted here this overhead is on the order of 10%.
- There is an inherent overhead in the interleave scheme (see the red area in Fig 5). This overhead decreases as we increase the number of slices.

Assuming instantaneous data transfer, the optimal run time of our hybrid implementation is equal to the time for the linear solver. Our implementation is, depending on the configuration, within 5% (double precision, single socket) to 25% (double precision, dual socket) of that value.

## 5 Intel Xeon Phi implementation

In essence the implementation on the Xeon Phi is similar to the GPU implementation. However, there are two major differences. First, due to the 512 bit wide vector units, vectorization is extremely important to obtain good performance on the Xeon Phi. In order to enable the compiler to generate efficient code for the assembly step, we have added __restrict and const keywords to our computational kernels. This is rather straightforward to do as the computational kernels are implemented using simple data structures and abstractions are only build on top of that layer. We have used the vectorization report of the Intel C compiler to check that the compiler has indeed sufficient information to vectorize the time intensive portions of our algorithm. This has to be contrasted with the GPU implementation of the assembly step which is relatively straightforward (neither warp divergence nor coalesced memory access is a major concern in this application). Note, however, that the code for the Intel Xeon Phi is essentially identical to the optimized code for the CPU.

**Fig 6:**
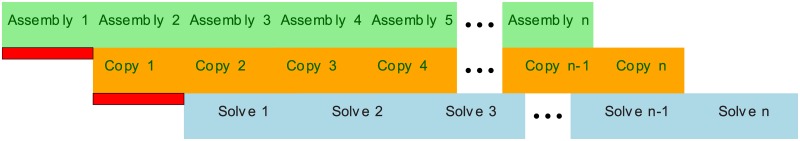
This figure shows a communication hiding pattern that interleaves the assembly (green; on the Xeon Phi), the copy (orange; data transfer from the Xeon Phi to the CPU), and the linear solve (blue; on the CPU). The red areas constitute the remaining overhead that decreases as we divide our problem into smaller and smaller slices.

Second, since the assembly step takes significantly longer on the Xeon Phi 7120 compared to the NVIDIA K80, it is no longer true that assembly (on the Xeon Phi) together with data transfer (from the Xeon Phi to the CPU) consumes less time than the linear solver (on the CPU). Thus, in order to obtain good performance we have to interleave all three operations as shown in Fig 6. All data transfer operations to and from the Xeon Phi are explicitly handled in the code. If this is not done a significant performance penalty is incurred. In order to avoid any overhead due to the quite expensive memory allocation on the Xeon Phi, the memory required for the computation is only allocated once (at the beginning of the simulation).

The timing results for the Xeon Phi 7120 are given in Table 4. We observe a speedup of approximately 2.5 (for both single and double precision) for adding a single Xeon Phi 7120 to the dual socket workstation. On the other hand, for a single socket workstation the observed speedup is approximately 3.2 (single precision) and 3.5 (double precision).

**Table 4:** Timing results for the hybrid algorithm (Xeon Phi 7120+CPU) illustrated in Fig 6. The wall time (W), the time required to assemble the system (A), the time required for the linear solves (L) and the overhead due to offloading to the Phi (O) is shown. Note that the overhead is defined such that W = L + O. The number of slices that yield the optimal run time are highlighted in bold in the table. All measured times are in units of seconds. In addition, the standard deviation determined from 20 repetitions of the simulation is shown next to the wall time.

**single precision**						
Hardware	slices	W	L	O	A	speedup
CPU	–	6.61±0.01	1.68	–	4.93	–
Phi+CPU	1	3.67±0.02	1.69	1.98	0.88	1.80
	5	2.31±0.02	1.71	0.59	0.99	2.87
	10	2.12±0.03	1.69	0.43	1.04	3.12
	15	2.15±0.03	1.75	0.39	1.27	3.08
	**20**	**2.09±0.04**	**1.75**	**0.34**	**1.08**	**3.16**
2xCPU	–	3.70±0.03	1.00	–	2.70	–
Phi+2xCPU	1	2.94±0.03	0.98	1.96	0.88	1.26
	5	1.61±0.04	1.01	0.5	0.99	2.30
	**10**	**1.47±0.05**	**1.05**	**0.42**	**1.03**	**2.52**
	15	1.59±0.05	1.09	0.49	1.27	2.33
	20	1.52±0.05	1.12	0.40	1.08	2.43
GPU+CPU	20	1.84	1.73	0.10	0.47	3.60
GPU+2xCPU	20	1.22	1.12	0.10	0.47	3.03
**double precision**						
Hardware	slices	W	L	O	A	speedup
CPU	–	12.05±0.01	2.80	–	9.26	–
Phi+CPU	1	6.76±0.03	2.84	3.92	1.80	1.78
	5	3.97±0.06	2.79	1.18	2.04	3.04
	10	3.60±0.07	2.78	0.82	2.15	3.35
	15	3.63±0.10	2.86	0.78	2.73	3.32
	**20**	**3.48±0.09**	**2.86**	**0.62**	**2.20**	**3.46**
2xCPU	–	7.01±0.01	1.91	–	5.11	–
Phi+2xCPU	1	5.87±0.02	1.92	3.95	1.80	1.20
	5	3.13±0.06	1.97	1.16	2.03	2.24
	**10**	**2.84±0.08**	**2.04**	**0.80**	**2.15**	**2.47**
	15	3.17±0.07	2.07	1.10	2.77	2.22
	20	2.91±0.09	2.12	0.79	2.17	2.41
GPU+CPU	15	2.99	2.77	0.22	0.78	4.03
GPU+2xCPU	15	2.44	2.20	0.24	0.78	2.88

Note that the performance of the GPU implementation on one-half of the NVIDIA K80 (considered in section 4) is superior by approximately 20% (for the dual socket case) and approximately 15% (for the single socket case) compared to the Xeon Phi 7120 implementation. We should also note that, as discussed before, the interleave scheme is out of necessity somewhat more complicated than the interleave scheme that is used for the GPU code (see Fig 5).

The performance difference between the Intel Xeon Phi 7120 and the NVIDIA K80 are mainly explained by the fact that the assembly step is more costly on the Xeon Phi 7120. Therefore, it is not possible to hide the data transfer as well as on the K80 which negatively impacts the performance of the implementation.

## 6 Multiple GPU implementation

The GPU implementation in section 4 uses a single GPU to perform the assembly step of the optimization algorithm. However, as has been pointed out in the introduction, the NVIDIA K80 includes two identical GPUs within the same expansion card. Thus, so far we have only used one half of the computational potential within that package. Certainly, we can not expect a factor of two improvement when using this additional GPU as in the present implementation performance is mainly limited by the linear solve conducted on the CPU. However, the timing results given in Table 2 suggest that we could solve part of the problem (both assembly and linear solve) on the second GPU. In this situation, optimal load balancing dictates the amount of work that is parceled out to the second GPU. Based on Table 2 we would expect that we achieve optimal performance by assigning 35% (double precision, single socket), 30% (double precision, dual socket and single precision, single socket), and 20% (single precision, dual socket) of the work set to the second GPU. Thus, in most situations we would expect a maximal speedup of about 40–50% (compared to the single GPU implementation). The exception being the single precision dual socket configuration in which a maximal speedup of only 25% is possible.

Since both the assembly step and the linear solve are computed on the second GPU, we first completely assemble the systems (using a single CUDA kernel call) and then perform the linear solves (using a single MAGMA call). In this process no data needs to be transferred to or from the GPU and we do not divide our parcel of the workload into slices. In fact, doing the latter incurs a small but significant performance penalty.

There is one additional issue that deserves our attention. While the MAGMA linear algebra library includes routines that use the GPU memory as input and output, it is primarily designed to operate in an environment that includes CPUs as well as GPUs. Consequently, there is no way to execute a MAGMA routine without CPU support and in an asynchronous fashion. To avoid oversubscription (which measurements show has a negative impact on performance) we use only 15 OpenMP threads for the linear solve and execute the MAGMA call in a separate pthread. However, it is clear that this reduces the maximal achievable improvement in performance to a certain degree.

The timing results for this implementation are shown in Table 5. We observe a speedup of 3.4 (single precision) and 3.8 (double precision) for adding a K80 to the dual socket workstation. In the case of a single socket workstation the observed speedup is approximately 4.7 (single precision) and 5.6 (double precision). We remark that the speedup compared to the single GPU implementation is in all cases within 5% of the maximal achievable speedup (based on the design decisions outlined in this section).

**Table 5:** Timing results for the hybrid algorithm (full NVIDIA K80+CPU) that uses both GPUs of the K80 hardware. The wall time (W), the time required to assemble the system (A), the time required for the linear solves (L) and the overhead due to offloading to the GPUs (O) is shown. Note that for the GPU implementation the time required by the linear solve (which is done on the CPU) always dominates the total runtime. Thus, we have W = L + O. The number of slices and work distribution that yields the optimal run time are highlighted in bold in the table. All measured times are in units of seconds. In addition, the standard deviation determined from 20 repetitions of the simulation is shown next to the wall time.

**single precision**						
Hardware	slices, distr	W	L	O	A	speedup
CPU	–	6.61±0.01	1.68	–	4.93	–
2xGPU+CPU	15, 0.35	1.57±0.01	1.26	0.31	0.45	4.21
	**15, 0.30**	**1.41±0.02**	**1.28**	**0.13**	**0.42**	**4.68**
	20, 0.30	1.42±0.02	1.29	0.13	0.42	4.65
	20, 0.25	1.48±0.02	1.36	0.12	0.35	4.47
2xCPU	–	3.70±0.03	1.00	–	2.70	–
2xGPU+2xCPU	15, 0.30	1.38±0.01	0.92	0.46	0.33	2.68
	15, 0.25	1.18±0.01	0.95	0.23	0.35	3.14
	**15, 0.20**	**1.08±0.02**	**0.98**	**0.11**	**0.37**	**3.41**
	20, 0.20	1.10±0.02	0.99	0.11	0.37	3.37
GPU+CPU	20	1.84	1.73	0.10	0.47	3.60
GPU+2xCPU	20	1.22	1.12	0.10	0.47	3.03
**double precision**						
Hardware	slices, distr	W	L	O	A	speedup
CPU	–	12.05±0.01	2.80	–	9.26	–
2xGPU+CPU	10, 0.40	2.31±0.01	1.79	0.52	0.47	5.23
	**10, 0.35**	**2.14±0.04**	**1.91**	**0.23**	**0.51**	**5.63**
	15, 0.35	2.15±0.03	1.94	0.22	0.51	5.60
	15, 0.30	2.30±0.03	2.09	0.21	0.55	5.23
2xCPU	–	7.01±0.01	1.91	–	5.11	–
2xGPU+2xCPU	10, 0.35	2.08±0.01	1.50	0.58	0.51	3.38
	10, 0.30	1.88±0.01	1.64	0.23	0.55	3.73
	**15, 0.30**	**1.86±0.02**	**1.63**	**0.23**	**0.55**	**3.78**
	15, 0.25	1.90±0.02	1.67	0.23	0.59	3.70
GPU+CPU	20	3.08	2.88	0.20	0.78	3.91
GPU+2xCPU	15	2.44	2.20	0.24	0.78	2.88

## 7 Conclusion

We have compared the speedup that can be achieved for a genetic optimization algorithm that uses a panel method as the inner solver when an Intel Xeon Phi 7120 or a NVIDIA K80 is added to a workstation with one or two Intel Xeon E5-2630 v3 CPUs. Optimization and parallelization for the CPU and Intel Xeon Phi code is done using the Intel C compiler (vectorization) and OpenMP. For the GPU we use an implementation that is based on CUDA. Since the linear solver is faster on the CPU and the assembly is faster on the Xeon Phi 7120/NVIDIA K80, the present algorithms profits from a hybrid implementation that uses both traditional CPUs as well as accelerators. The obtained results can be summarized as follows:

- Adding a K80 to the dual socket workstation results in a speedup of approximately 3.4 (single precision) and 3.8 (double precision).
- Adding a Xeon Phi 7120 to the dual socket workstation results in a speedup of approximately 2.4 (single precision) and 2.5 (double precision).
- Since the performance of the CPU only implementation is mostly dominated by the assembly step, the speedups for a single CPU are significantly larger. In this configuration we observe speedups of up to 5.6 on the NVIDIA K80 and up to 3.5 for the Xeon Phi 7120 implementation.

These speedups are clearly of practical interest. This is true both for the NVIDIA K80 as well as for the Xeon Phi 7120. For the problem under consideration the NVIDIA K80 yields better performance compared to the Xeon Phi 7120. What is not so clear cut is the development effort that is required for each platform. One advantage of the Xeon Phi is that once we had an optimized code for the assembly step on the CPU (using vectorization and OpenMP) we almost immediately obtained good performance on the Xeon Phi. On the other hand, the CUDA implementation of the assembly step is straightforward and due to the computational advantage of the GPU a less complicated communication hiding scheme proves sufficient. Thus, with respect to the development effort involved there is no clear winner.
